# Assessing the Potential Apoptotic Effects of Different Hydatid Cyst Fluids on Human Healthy Hepatocytes and Hepatocellular Carcinoma Cells

**DOI:** 10.1007/s11686-024-00797-z

**Published:** 2024-02-19

**Authors:** İpek Baysal, Serra Örsten, Görkem Cengiz, Emre Ünal, Ahmet Bülent Doğrul, Türkmen Çiftçi, Samiye Yabanoğlu Çiftçi, Devrim Akinci, Okan Akhan

**Affiliations:** 1 Vocational School of Health Services, Hacettepe University, 06100 Ankara, Turkey; 2 Vocational School of Health Services, Yüksek İhtisas University, 06291 Ankara, Turkey; 3 Faculty of Medicine, Department of Radiology, Hacettepe University, 06100 Ankara, Turkey; 4 Faculty of Medicine, Department of General Surgery, Hacettepe University, 06100 Ankara, Turkey; 5 Faculty of Pharmacy, Department of Biochemistry, Hacettepe University, 06100 Ankara, Turkey

**Keywords:** *Echinococcus Granulosus*, Hydatid cyst fluid, Cancer, Apoptosis, Anti-cancer Effect

## Abstract

Cystic Echinococcosis (CE) is a zoonotic infection caused by the larval form of *Echinococcus granulosus* in humans. Emerging evidence suggests an intriguing inverse association between *E. granulosus* infection and the occurrence of cancer. This study aimed to investigate the influence of diverse host-derived hydatid cyst fluids (HCF) with distinct genotypes on human liver hepatocytes (HC) and hepatocellular carcinoma cells (HepG2). Specifically, we examined their effects on cell proliferation, apoptosis sensitivity (BAX/BCL-2), apoptosis-related p53 expression, and the expression of cancer-related microRNA (hsa-miR-181b-3p). Cell proliferation assays, real-time PCR, and ELISA studies were conducted to evaluate potential anti-cancer properties. The findings revealed that animal-origin HCF (G1(A)) induced direct cell death by augmenting the susceptibility of HepG2 cells to apoptosis. Treatment with both G1(A) and G1(H) HCF sensitized HepG2 and HC cell lines to apoptosis by modulating the BAX/BCL-2 ratio, accompanied by upregulation of the p53 gene. Additionally, G1(A) HCF and human-derived HCFs (G1(H), G7(H)) reduced the expression of miR-181b-3p in HepG2 cells. Consequently, this study demonstrates the potential anti-cancer effect of HCF in HepG2 cells and provides the first comparative assessment of HCFs from human and animal sources with diverse genotypes, offering novel insights into this field.

## Introduction

Evidence shows that various parasitic diseases can trigger anticancer processes in the relationship between parasites and their hosts [1]. Previous studies have supported that certain parasites including the protozoans such as *Trypanosoma cruzi*, *Toxoplasma gondii*, and *Acanthamoeba castellanii*, and the helminths as *Echinococcus granulosus* and *Strongyloides stercoralis* exhibit anticancer activities *in-vitro* [2–6].

Cystic echinococcosis (CE) is a neglected zoonotic infection in intermediate hosts and humans caused by the larval form of *Echinococcus granulosus* sensu lato [7]. *E. granulosus* s.l. is defined as a species complex that includes *Echinococcus granulosus* sensu stricto (s.s.) (genotypes G1-G3), *Echinococcus equinus* (G4 genotype), *Echinococcus ortleppi* (G5 genotype), *Echinococcus canadensis* cluster (G6-G8, G10 genotypes) and *Echinococcus felidis* [8–11]. Although CE has a cosmopolitan distribution, it is frequently seen in the Mediterranean, Asia and South America [12, 13]. The disease always has an asymptomatic onset and may remain silent for years [7]. In symptomatic cases, on the other hand, although clinical symptoms vary, they are not specific to the disease [14].

Although there are conflicting reports about the relationship of *E.granulosus* with cancer, most studies argue that it reduces the development of cancer [1]. A retrospective study conducted in Turkey reported that the prevalence of cancer in CE patients was significantly lower [15]. Though there is a pilot-retrospective study from Cyprus with the direct opposite result [16], more findings support the concept that *E. granulosus* reduces cancer growth directly or indirectly [1]. In the literature, it has been suggested that the hydatid cyst fluid (HCF) can be used against cancer as an immunotherapeutic agent [17]. However, the underlying molecular mechanisms are still unclear.

The main aim of this study was to investigate and assess the impact of administering various host-derived HCFs from different genotypes, obtained from both humans and animals, on cell proliferation, apoptosis sensitivity (measured by BAX/BCL-2 protein levels), and p53 gene expressions in human healthy liver hepatocytes and human hepatocellular carcinoma cells. Additionally, the study sought to examine the effects of HCF treatment on miR-181b-3b, a molecule associated with hepatocellular carcinoma, in the cells treated with HCFs, and to determine whether the anti-cancer effect of hydatid cyst fluids differs among genetically distinct species.

## Materials & Methods

### Ethics Statement

This study was approved by the Institutional Ethical Committee of the Faculty of Medicine, Hacettepe University (2021/14–61).

### Hydatid Cyst Fluid Collection and Molecular Characterization

Human derived-HCFs were collected from patients who underwent percutaneous treatment in the interventional radiology unit from September 2021 to January 2022. Animal-derived HCFs were collected during postmortem examination of the internal organs of sheep for the presence of CE by inspection, palpation and incision from the slaughterhouses of Ankara provinces between September 2021- December 2021.

For direct microscopy, HCF was centrifuged at 4000 rpm for 15 min and the pellet was examined under a light microscope at × 40 objective. The presence of hook or protoscoleces’ on microscopy was accepted as an indicator of cyst fertility. In order to select HCFs belonging to different genotypes, genetic characterization of *E.granulosus* was performed. Briefly, DNA was extracted from HCF using the DNA Extraction Kit (GeneAll Biotechnology, Korea) according to the manufacturer’s instructions. To amplify a partial fragment of the cytochrome c oxidase subunit 1 (mt-CO1) mitochondrial gene, the PCR was performed as previously described [18]. As a result of gel electrophoresis, all amplicons considered positive were characterized by sequencing to select genetically different HCFs. The sequence data was evaluated using the BLAST algorithm (http://www.ncbi.nlm.nih.gov/BLAST/). According to sequence analysis, the animal-derived isolates (A) were identified as *E.granulosus* s.s. (G1-G3 genotypes). Additionally, among the human-derived isolates (H), *E.granulosus* s.s. (G1-G3 genotypes) and *E.canadensis* (G6-G7 genotypes) were selected. Hence, the aforementioned genotypes were used in the treatment of cells. All HCFs used for application were fertile. None of the administered HCFs were obtained from infected cysts. The main aim of the investigation was to determine the anti-cancer effect of the antigens in the fluid; therefore, no sterilization process was applied.

### Cell Culture Studies

Human liver hepatocyte cell line (HC) (AcceGen, United States) and human hepatocellular carcinoma (HepG2) (ATCC, United States) cell line were used in the study. HepG2 and HC cell lines were suspended in DMEM/F12 medium containing 10% fetal bovine serum (FBS), supplemented with antibiotics (100 U/ml penicillin, 100 lg/ml streptomycin) (complete medium). Cells were grown under standard culture conditions (37 °C and 5% CO_2_) in a humidified incubator.

### Cell Proliferation Assay (XTT)

To assess the impact of various host-derived hydatid cyst fluids (HCFs) (G1(A), G1(H), G7(H)) on the proliferation of HC and HepG2 cells, an XTT assay was conducted. Approximately 5 × 10^4^ cells were seeded in individual wells of 96-well cell culture plates containing complete medium. The cells were then treated with different dilutions (1:3, 1:2) of hydatid cyst fluids, which were diluted with PBS. The treatment was carried out without changing the volume of the medium, and the plates were subsequently incubated at 37 °C for a period of 24 h. Following the incubation period, 50 µL of XTT solution was added to each well, and the cells were further incubated at 37 °C for 2 h. Subsequently, the absorbance of each well was measured using a microplate reader (FLUOstar® Omega, BMG LabTech, Germany) at a wavelength of 450 nm. The obtained results were normalized using the values from the control group and expressed as a percentage of viability. (*n* = 6).

### Real-Time Polymerase Chain Reaction (RT-PCR)

HC and HepG2 cells were seeded in 6-well plates at approximately 3 × 10^5^ cells/well in complete medium and after reaching full confluency, cells were treated with HCFs (G1(A), G1(H), G7(H)) at different dilution ratios (1:3, 1:2) and cells were incubated at 37 °C for 24 h. Following the application procedure, total RNA was isolated from the cells with the extraction kit (GeneAll Biotechnology, Korea) according to the manufacturer’s instructions. cDNA was synthesized from 1 µg of extracted total RNA according to the manufacturer’s protocol (Applied Biosystems, United States) and aliquots stored at -20 °C. The absence of contamination in the samples was confirmed by negative control samples prepared without reverse transcriptase. RT-PCR analysis was carried out using a ViiA7 Sequence Detection System (Applied Biosystems) in 96-well plates. The PCR reactions were performed in triplicate with cDNA, primers, PCR Master Mix containing dNTPs, polymerase, and SYBR green. The PCR cycling conditions involved a single cycle of 94 °C for 10 min, followed by 40 cycles of 95 °C for 15 s, annealing, and 60 °C for 1 min. After each application, the fold change of the investigated gene was normalized by dividing the value of the internal control gene. Hsa-miR16-5p was used as an internal control gene [19] for miRNA expression (*n* = 4). The comparative Ct (∆∆CT) method for quantification was used for the relative expression of selected miRNAs. The sequences of the forward and reverse primer pairs used are shown in Table 1.

**Table 1:** Primer sequences used in RT-PCR experiment

Primer	Sequence
Hsa-miR181b-3p	F: 5′-CTCACTGAACAATGAATGCAA-3′
Hsa-miR16-5p	F:5′-ACACTCCAGCTGGGTAGCAGCACGTAAATATTGGC-3′
Universal Reverse (miRNA)	R: 5′-TGGTGTCGTGGAGTCG-3′
GAPDH	F: 5′-CCATGGGGAAGGTGAAGGTC-3′ R: 5′-AGTGATGGCATGGACTGTGG-3′
p53	F: 5′-CAG CAC ATG ACG GAG GTT GT-3′ R: 5′-TCA TCC AAA TAC TCC ACA CGC-3′

### ELISA Analyzes

HC and HepG2 cells were seeded in 6-well plates at approximately 3 × 10^5^ cells/well in complete medium. After 24 h of incubation, cells were treated with HCFs (G1(A), G1(H), G7(H)), at different dilution ratios (1:3, 1:2) and were incubated at 37 °C for 24 h. Following the treatment, cells were collected in accordance with the kit instructions cells; BAX and BCL-2 (Bioassay Technology Laboratory, China) protein levels were measured (*n* = 4). Briefly, the standards were diluted using a standard dilution buffer through serial dilution. Subsequently, the samples and the standards were added to 96-well plates provided in the kit. Then, 10 µL of antibodies were added to the samples and 50 µL of streptavidin-HRP was added to each well. The plate was covered and incubated at room temperature at 37 °C for an hour. After the incubation, the wells were washed four times with a washing solution, and 50 µL of substrate A and 50 µL of substrate B were added to all wells, respectively. Following a 10-minute incubation in the dark at room temperature, 50 µL of stop solution was added, and the blue color turned yellow. Finally, the absorbance was measured at 450 nm using an ELISA reader (BMG LabTech, Germany).

### Statistical Analysis

GraphPad Prism 8.4.3 software was used for the statistical analyses. The results were interpreted with two way-ANOVA. A *p*-value of 0.05 or lower was considered as statistically significant.

## Results

### Cell Proliferation Assay (XTT)

**Fig. 1:**
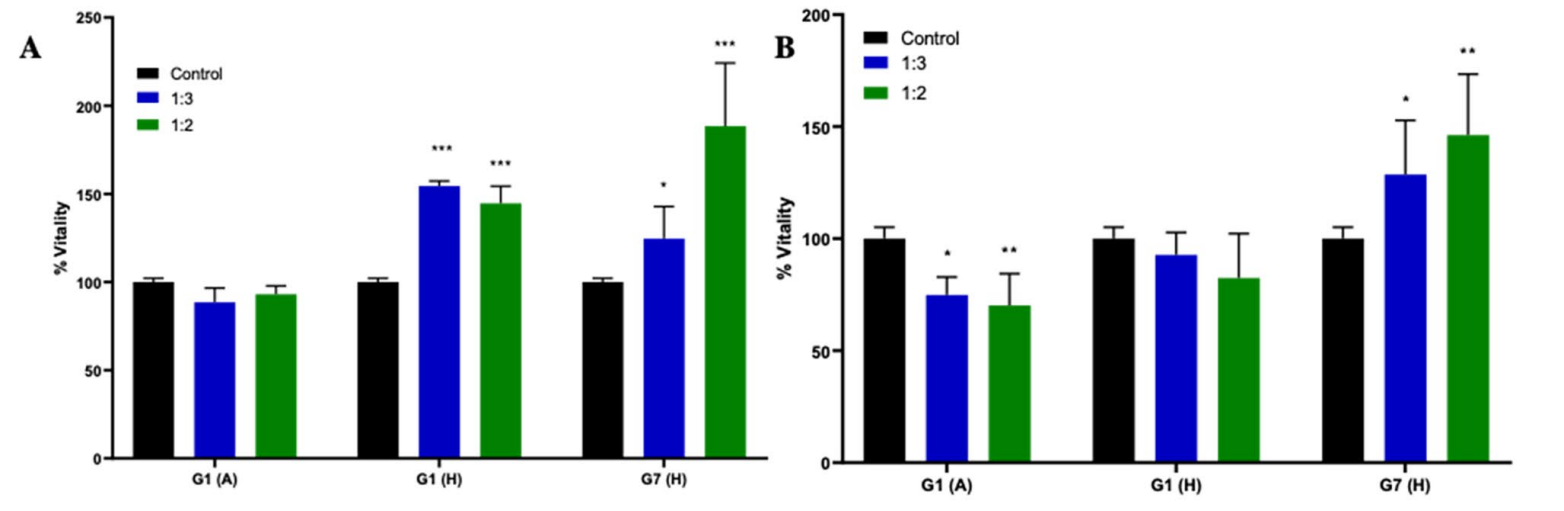
Effect of HCF on cell viability in (**A**) HC and (**B**) HepG2 cells (*P < 0.05, **P < 0.01, ***P < 0.001 vs. control)

XTT test was performed to determine and compare the effects of HCFs (G1(A), G1(H), G7(H)) on the proliferation of HepG2 and HC cells. The XTT test results are given in Fig. 1.

**Fig. 2:**
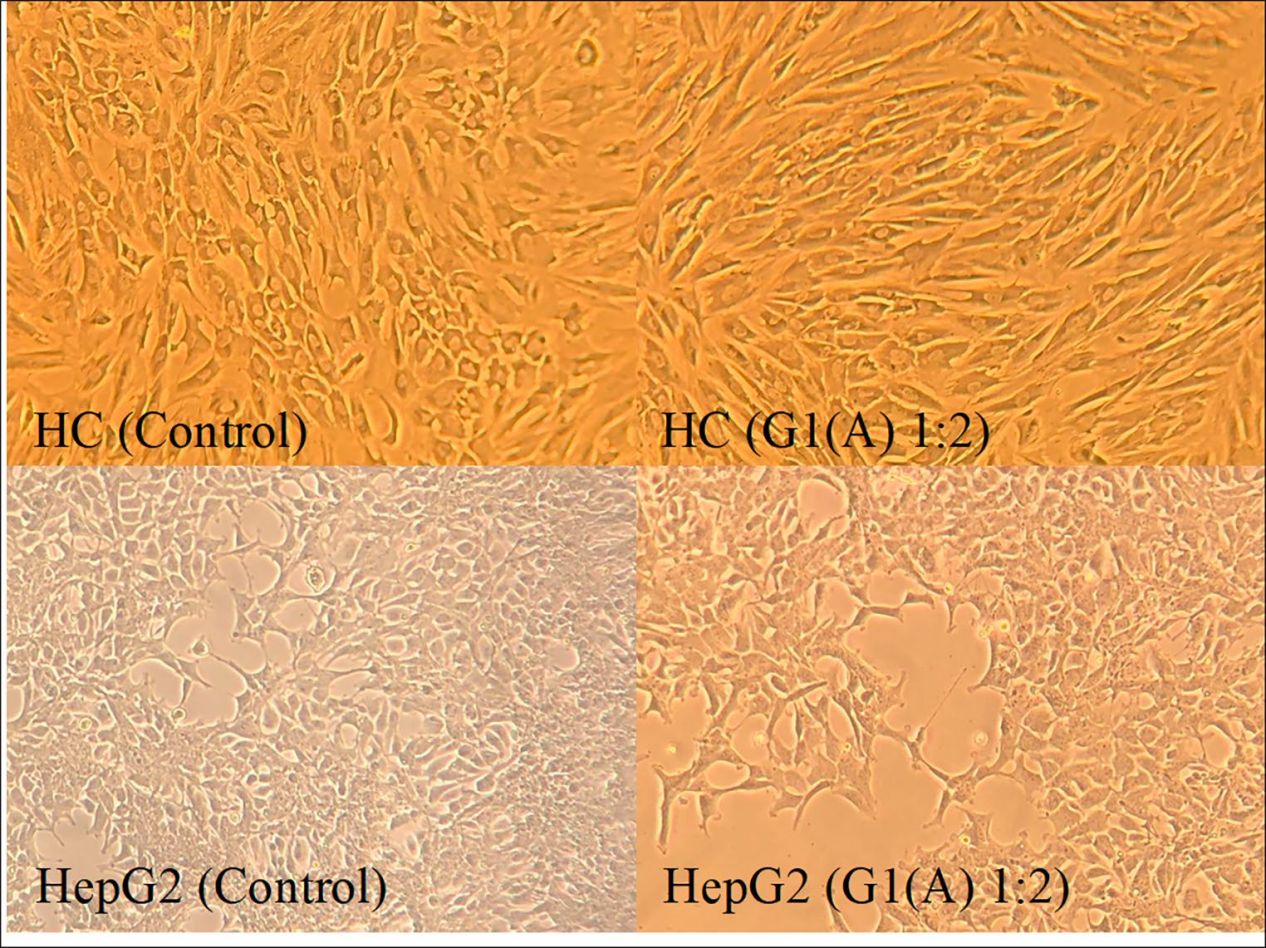
Images of G1(A) administration on HC and HepG2 cell lines

In HC cells, the administration of G1(A)-HCF did not show a significant impact on cell viability. However, both G1(H) and G7(H) HCFs increased cell viability (*P* < 0.001 and *P* < 0.05, respectively). On the other hand, when considering HepG2 cell lines, G1(H)-HCF did not have a significant effect on cell viability, while G7(H)-HCF increased cell viability (*P* < 0.05). Interestingly, the administration of G1(A)-HCF resulted in a decrease in cell viability (*P* < 0.05) (Fig. 2).

### RT-PCR Results

**Fig. 3:**
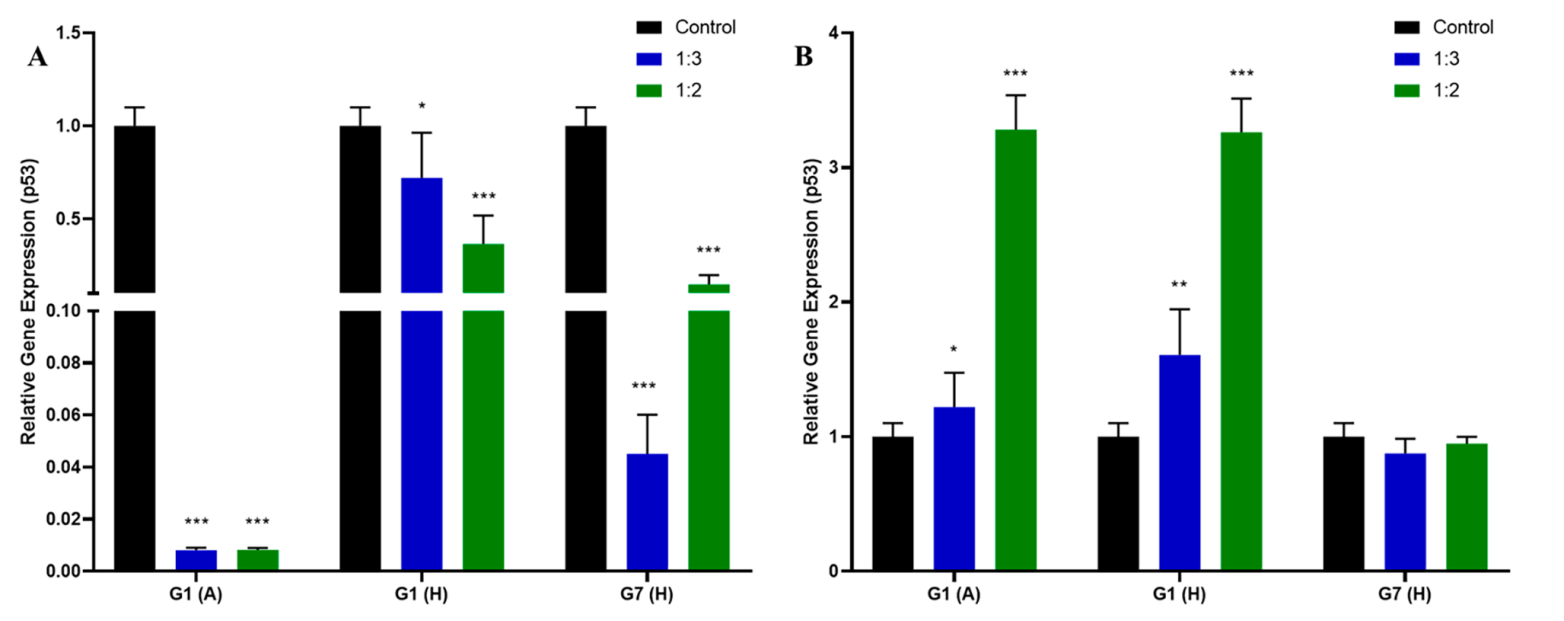
P53 expression in (**A**) HC and (**B**) HepG2 cells after HCF administration (*P < 0.05, **P < 0.01, ***P < 0.001 vs. control)

**Fig. 4:**
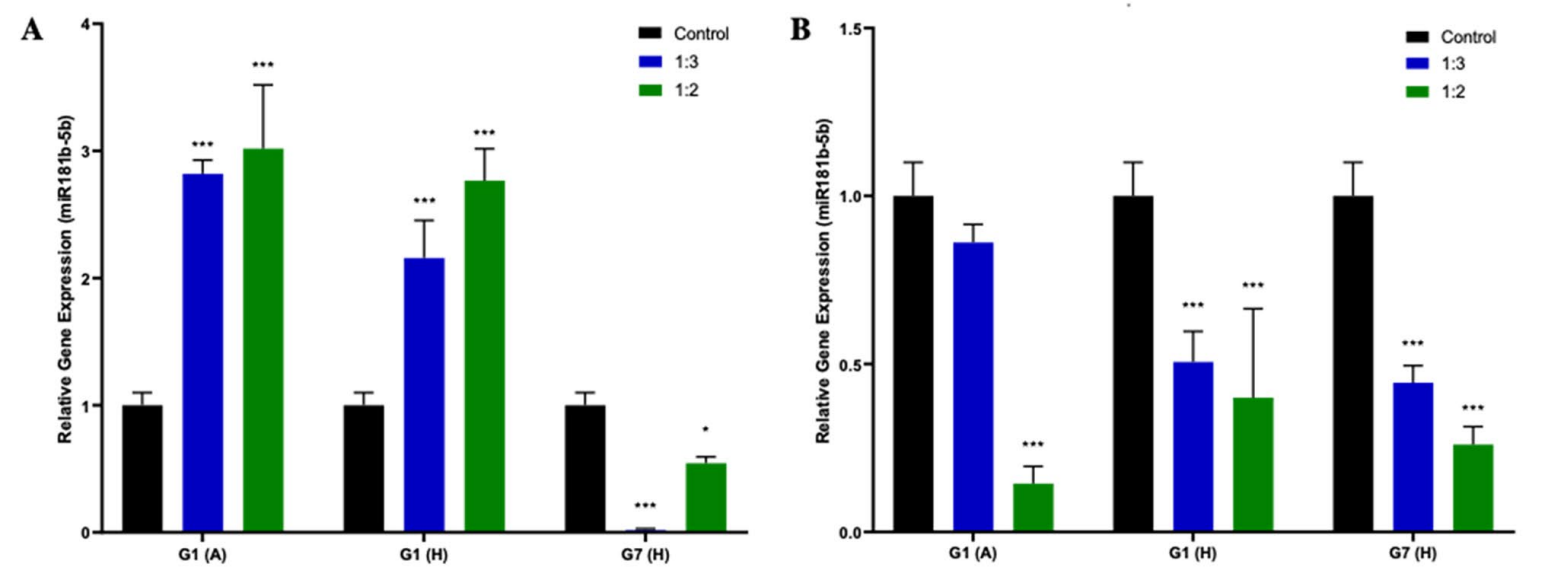
Hsa-miR181b-3p expression in (**A**) HC and (**B**) HepG2 cells after HCF administration (*P < 0.05, **P < 0.01, ***P < 0.001 vs. control)

Within the scope of the study, the anti-cancer effects of HCFs in HepG2 and HC cells were examined and compared. To assess these effects, the expression levels of the apoptosis-related gene p53 and the hepatocellular carcinoma-associated microRNA (hsa-miR-181b-3p) were analyzed using RT-PCR. p53 and hsa-miR-181b-3p expression results are given in Figs. 3 and 4, respectively.

When analyzing the results of the p53 gene in HC cells, all treatments led to a significant decrease in expression levels (*P* < 0.001), irrespective of the concentration. However, in the case of HepG2 cells, administration of G1(A) and G1(H) resulted in an increase in expression levels (*P* < 0.001), while the administration of G7(H) did not have a statistically significant effect on expression levels.

Furthermore, the study investigated the impact of various HCFs on the expression of hsa-miR-181b-3p, a molecule associated with hepatocellular carcinoma, in both HC and HepG2 cells. In HC cells, the administration of G1(A) and G1(H) HCFs resulted in a significant increase in expression levels (*P* < 0.001), whereas the administration of G7(H) HCF led to a significant decrease in expression levels (*P* < 0.05). Conversely, in HepG2 cells, all HCF administrations significantly reduced the expression levels (*P* < 0.001).

### ELISA Results

**Fig. 5:**
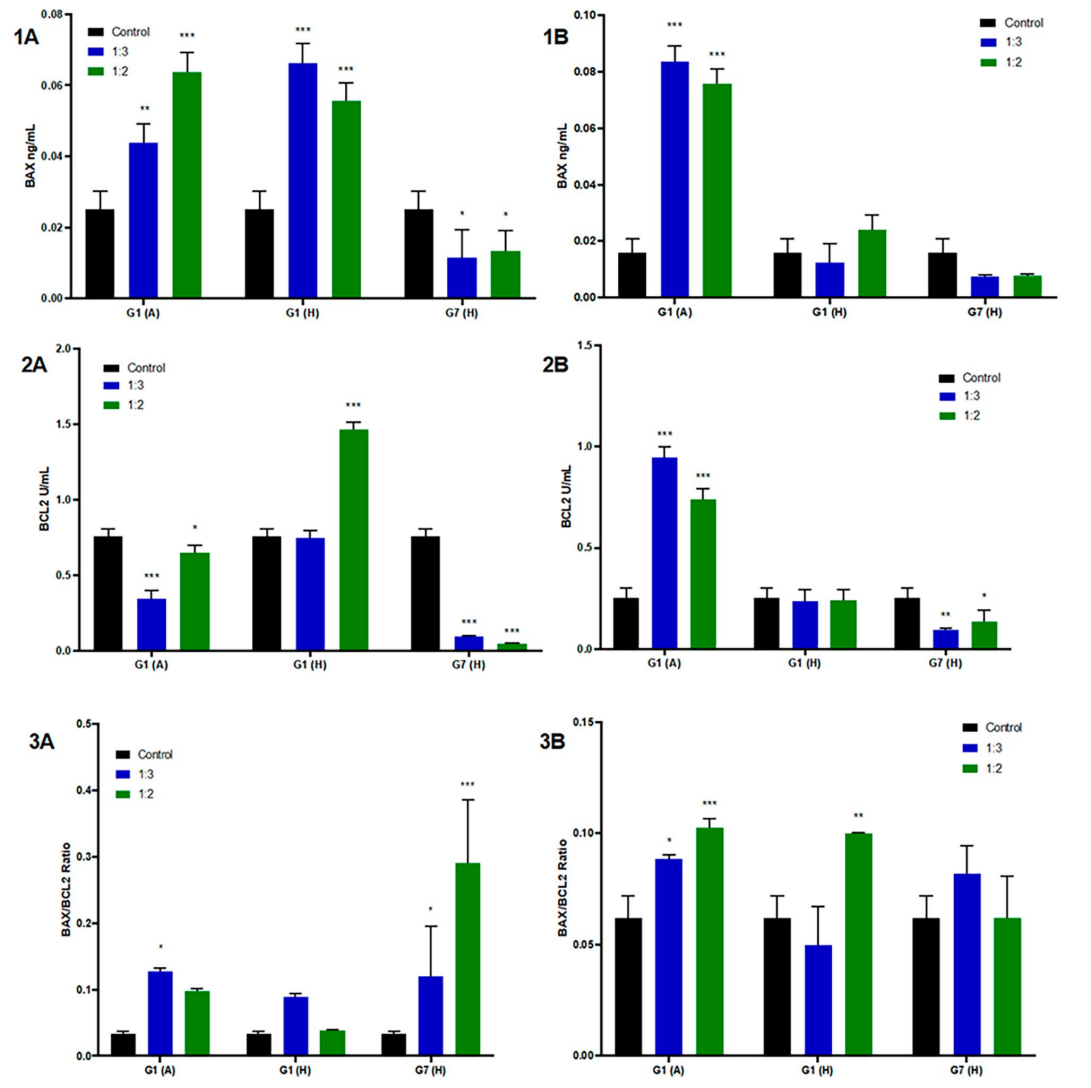
BAX (1), BCL-2 (2) protein levels and BAX/BCL-2 (3) ratio in (**A**) HC and (**B**) HepG2 cell lines after administration of HCFs (*P < 0.05, **P < 0.01, ***P < 0.001 vs. control)

In order to investigate the anti-cancer effect of HCFs on HepG2 and HC cells in addition to gene expression studies, the expression levels of BAX and BCL-2 proteins were also measured and BAX / BCL-2 ratio was determined. The results of BAX and BCL-2 protein levels and BAX/BCL-2 ratio obtained after treatment of HC and HepG2 cells with HCFs are given in Fig. 5.

In HC cells, the administration of G1(A) and G1(H) HCFs resulted in a significant increase in BAX protein levels (*P* < 0.001), while G7(H) HCF administration led to a significant decrease in protein levels (*P* < 0.001). However, in HepG2 cells, the BAX protein levels showed a significant increase only after G1(A) HCF administration (*P* < 0.001). No statistically significant changes in BAX protein expression were observed following G1(H) and G7(H) HCF administrations (see Figs. 5-1A, 1B).

In HC cells, the results showed a decrease in BCL-2 protein expression after the administration of G1(A) and G7(H) (*P* < 0.05), while an increase was observed after the administration of G1(H) (*P* < 0.001). On the other hand, in HepG2 cells, the administration of G1(A) resulted in an increase (*P* < 0.001) in BCL-2 protein expression levels, while the administration of G7(H) led to a decrease (*P* < 0.05). However, the expression levels of BCL-2 protein were not found to be statistically affected after the administration of G1(H) in HepG2 cells (Figs. 5-2A, 2B).

Following HCF treatment, the BAX/BCL-2 ratio was calculated as an indicator of cell sensitivity to apoptosis. Protein levels were used to determine this ratio. In HC cells, the BAX/BCL-2 ratio increased significantly after G1(H) and G7(H) administrations (*P* < 0.05), indicating a higher sensitivity of these cells to apoptosis. Conversely, in HepG2 cells, the BAX/BCL-2 ratio increased significantly after the administration of G1(A) (*P* < 0.001) and G1(H) (*P* < 0.01), suggesting a greater apoptotic response of these cells to these specific treatments. These findings demonstrate that the effects of the various treatments on the BAX/BCL-2 ratio differed between the two cell types, highlighting their distinct impacts on the apoptotic response (refer to Figs. 5-3A, 3B).

## Discussion

This study aimed to investigate the anti-cancer properties of various host-derived HCFs with different genotypes on HepG2 and HC cells. Although there are conflicting reports in the literature regarding the relationship between * E. granulosus* and cancer, most available data suggest that *E. granulosus* may have an anti-cancer effect. Since the liver is commonly affected by CE, human liver cells were chosen for this study. The impact of HCF administration on BAX and BCL-2 protein levels and p53 gene expression in HC and HepG2 cells was examined to explore the anti-cancer effect through apoptotic mechanisms. The BCL-2 family consists of different members with distinct roles, including promoting cell survival (such as BCL-2, BCL-HL, and MCL-1) or facilitating cell death (such as BAX, BAK, and BCL-XS). The p53 gene is capable of regulating cell susceptibility to apoptosis by downregulating BCL-2 expression and upregulating BAX expression. BAX and BCL-2 are well-known key regulators of the apoptotic process [20]. Thus, this study examined the factors involved in the apoptotic mechanism, as well as hsa-miR-181b-3p, a molecule associated with hepatocellular carcinoma. The effects of different HCF administrations were compared and analyzed.

HC and HepG2 cells were subjected to different ratios of host-derived HCFs (G1(A), G1(H), G7(H)), and their viability was evaluated using an XTT assay. Treatment with G1(A) did not result in a statistically significant change in HC cell viability, whereas a significant decrease in viability was observed in HepG2 cells. This observation is consistent with the findings on p53 gene expression. In HC cells, G1(A) administrations led to a statistically significant decrease in p53 gene expression, promoting cell survival and increased proliferation. In contrast, G1(A) treatment in HepG2 cells increased p53 gene expression, inducing apoptosis. These results support a recent study that reported a decreasing trend in the viability of A375 melanoma cancer cells with increasing concentrations of fertile HCF in an *in-vitro* cytotoxicity assay [6]. However, another study demonstrated no effect of animal-derived HCF on cell proliferation in human healthy lung epithelial (BEAS-2B) and human lung adenocarcinoma (A549) cell lines in vitro [21]. On the contrary, administration of G1(H) and G7(H) led to increased HC cell viability across all ratios (*P* < 0.001), in line with the observed decrease in p53 gene expression. However, G1(H) treatment did not significantly affect HepG2 cells, despite the observed increase in p53 gene expression levels. This discrepancy could be due to the relatively short 24-hour incubation period, which may not have been sufficient to fully manifest the effects of increased p53 gene expression in HepG2 cells, such as apoptosis. Surprisingly, G7(H) treatment unexpectedly resulted in increased cell viability of HepG2 cells (*P* < 0.05), even though the levels of p53 gene remained unchanged. Notably, proteomic studies have revealed the similarity between the host proteins present in HCF and those found in host plasma [22–24]. Host proteins that are defined in HCF are plasma proteins that mainly contain albumin, transferrin and various complement proteins [25, 26] [25, 26]. Our findings suggest that the host proteins present in human-derived HCFs may act as an additional nutrient source for the cells, potentially explaining the observed increase in cell viability when human-derived HCFs were administered to both HC and HepG2 cells. Moreover, our findings draw attention to noteworthy distinctions in the content levels of specific proteins across various genotypes. The observed variability underscores the potential role of genotype-specific protein expression in influencing cellular responses. Furthermore, the decrease in viability observed with the use of animal-derived HCF in our study aligns with previous research conducted on different cancer cell lines. These collective results provide valuable insights into the impact of host proteins in HCFs and their influence on cell viability [3, 27, 28].

The BAX/BCL-2 ratio was calculated following the administration of HCFs. The BAX and BCL-2 proteins have a clear antagonistic effect in regulating apoptosis, and the intracellular ratio of BAX/BCL-2 is considered a cellular marker of apoptosis susceptibility. In line with this concept, a cell with a high BAX/BCL-2 ratio is more responsive to apoptotic signals and more sensitive to various stimuli that induce cell death, including chemotherapeutic drugs, radiation, and hypoxia [29]. The impact of different host-derived HCF administrations on the BAX/BCL-2 ratio in HC and HepG2 cells was also evaluated. In HC cells, we observed that the BAX/BCL-2 ratio tended to increase with G1(A) and G7(H) administrations, while no significant change was observed with G1(H) administration. In HepG2 cells, G1(A) and G1(H) administrations significantly increased the BAX/BCL-2 ratio, while no significant change was observed with G7(H) administration. Thus, G1(A) and G1(H) administrations were found to have a higher anti-cancer effect when compared with G7(H). Contrary to our result, a recent study has shown that the *E. granulosus* PSCs did not induce significant differentiation in the morphology of HCC cells, but their proliferation and pathological properties were more altered after co-culturing with different quantities of PSCs *in-vitro.* Although they concluded that the mechanisms involved in the promoting effects of *E. granulosus* PSCs on proliferation, migration and invasion capacities of HCC cells, further investigation on the molecular mechanisms of processes is required [30]. The inconsistent results between studies may have been due to the fact that the whole HCF was used in our study and they only used PSCs.

A study investigating the miRNA expression profile differences between CE patients and healthy controls revealed that the identified miRNAs were related to cell proliferation, apoptosis, cell-cell interactions, and the cell cycle. The decrease in the expression of these miRNAs in CE patients suggested that CE may play a regulatory role in the anti-cancer effect through miRNA regulation [31]. To date, many mechanisms have been suggested for CE-related anti-cancer effects, including parasite molecules and activation of host immune response [1].

Additionally, hsa-miR-181b-3p expression, which is known to be associated with hepatocellular carcinoma, was also evaluated in HC and HepG2 cells before and after HCF administration. MiR-181b is transcriptionally activated by the Wnt/β-catenin signaling pathway in HCC [32]. A recent study reported that miR-181b expression was deregulated in the early stages of hepatocarcinogenesis induced by a diet containing choline and amino acids in C57BL/6 mice, and miR-181b was also shown to be significantly increased in the livers of mice that persisted to the preneoplastic stage. In conclusion, it was observed that TIMP3, which is a tumor suppressor and a target of miR-181b, caused significant suppression in the liver. In contrast, miR-181b increased the activity of MMP2 and 9, causing it to promote growth, clonogenic survival, migration, and increase of cells in HCC. It has also been shown that miR-181b is involved in the TGFβ signaling pathway [33]. Overexpression of miR-181b has also been found to increase the resistance of cells in HCC to doxorubicin, and an increase in miR-181b expression in patients with nonalcoholic steatohepatitis and HCC [33]. It has also been recently shown that miR-181b is overexpressed in the serum of patients with liver cirrhosis and targets the p27-regulated cell cycle [34]. These results suggest that miR-181b may play an important role in hepatocarcinogenesis. Based on the obtained results, we observed increased expression in HC cells after the administration of G1(A) and G1(H), whereas decreased expression was observed in HepG2 cells. In the case of G7(H) administration, a decrease in expression was observed in both HC and HepG2 cells. These findings suggest that the administration of HCF may lead to a decrease in the expression of hsa-miR-181b-3p and potentially influence hepatocarcinogenesis in HepG2 cells. However, further studies are required to fully comprehend the mechanism of action of hsa-miR-181b-3p in cancer and its interaction with HCF. Additionally, our findings indicate that the effects of HCFs from different hosts and genotypes vary. This variability could be attributed to differences in parasite protein concentrations within HCF and the presence of host proteins in the cyst fluids obtained from different hosts [24]. In addition, previous studies showed that *E.canadensis* (G6-G7 genotypes) exhibits clinically different features from *E.granulosus* s.s. (G1-G3 genotypes) [35–37]. Thus, it is not unexpected to observe variations in the anti-cancer effects between the two subspecies. Hence, the noticeable discrepancies in miR-181b expression highlight the intricate responses manifested by distinct genotypes within this experimental framework, might indicate a genotype-specific influence on miRNA regulation. Although our study provides valuable insights into the cellular responses to HCF in vitro, it is important to note that the translation of these findings to clinical applications may be limited. This limitation arises from the inherent differences between the controlled in vitro environment and the complex *in vivo* microenvironment. Recognizing this gap, further research is warranted to bridge these differences and better understand the clinical relevance of our observations.

The study has certain other limitations that should be acknowledged. One major limitation is the inability to analyze the HCFs at the molecular level, which hinders a comprehensive understanding of the specific molecules and mechanisms underlying the observed anti-cancer effect. Furthermore, another limitation is incomplete analysis of the anti-cancer effect at the gene and protein levels, providing a less comprehensive understanding of the molecular mechanisms driving these genotype-specific responses. These limitations highlight the need for further research to explore the molecular aspects and conduct more comprehensive analyses in order to gain a deeper understanding of the anti-cancer properties of HCFs.

In conclusion, this study conducted a novel evaluation and comparison of the anti-cancer effects of HCFs derived from various genotypes of human and animal sources on healthy hepatocytes and hepatocellular cancer cells. The assessment focused on the apoptotic pathway and miR-181b-5p expression. The results obtained from this study offer fresh insights into the potential utilization of HCFs with diverse genotypes for the development of innovative approaches in the treatment of hepatocellular cancer.

## Data Availability

The datasets generated during and/or analyzed during the current study are available from the corresponding author upon reasonable request.

## References

[1] Ranasinghe SL, McManus DP (2018) Echinococcus granulosus: Cure for Cancer Revisited. Front Med (Lausanne) 5 60. 10.3389/fmed.2018.00060 10.3389/fmed.2018.00060 PMC5857532 29594121

[2] Atayde VD, Jasiulionis MG, Cortez M, Yoshida N (2008). A recombinant protein based on Trypanosoma Cruzi surface molecule gp82 induces apoptotic cell death in melanoma cells. Melanoma Res.

[3] Yousofi Darani H, Soozangar N, Khorami S, Taji F, Yousofi M, Shirzad H (2012) Hydatid Cyst Protoscolices Induce Cell Death in WEHI-164 Fibrosarcoma cells and inhibit the proliferation of baby Hamster kidney fibroblasts in Vitro. J Parasitol Res 2012(304183). 10.1155/2012/304183 10.1155/2012/304183 PMC3306915 22496957

[4] Pidherney MS, Alizadeh H, Stewart GL, McCulley JP, Niederkorn JY (1993) In vitro and in vivo tumoricidal properties of a pathogenic/free-living amoeba. Cancer Lett 72(1–2) 91– 8. 10.1016/0304-3835(93)90016-3 10.1016/0304-3835(93)90016-3 8402581

[5] Plumelle Y, Gonin C, Edouard A, Bucher BJ, Thomas L, Brebion A, Panelatti G (1997). Effect of Strongyloides stercoralis infection and eosinophilia on age at onset and prognosis of adult T-cell leukemia. Am J Clin Pathol.

[6] Mohammadi M, Spotin A, Mahami-Oskouei M, Shanehbandi D, Ahmadpour E, Casulli A, Rostami A, Baghbanzadeh A, Asadi M (2021). MicroRNA-365 promotes apoptosis in human melanoma cell A375 treated with hydatid cyst fluid of Echinococcus granulosus sensu stricto. Microb Pathog.

[7] Eckert J, Gemmell MA, Meslin Fo-X, Pawlowski ZS, World Health O, WHO/OIE manual on echinococcosis in humans and animals: a public health problem of global concern / edited by J. Eckert… [et al.], Paris, France: World Organisation for Animal Health, 2001.

[8] Hüttner M, Nakao M, Wassermann T, Siefert L, Boomker JD, Dinkel A, Sako Y, Mackenstedt U, Romig T, Ito A (2008). Genetic characterization and phylogenetic position of Echinococcus Felidis (Cestoda: Taeniidae) from the African lion. Int J Parasitol.

[9] Lymbery AJ (2017). Phylogenetic pattern, evolutionary processes and species Delimitation in the Genus Echinococcus. Adv Parasitol.

[10] Romig T, Ebi D, Wassermann M (2015). Taxonomy and molecular epidemiology of Echinococcus Granulosus Sensu Lato. Vet Parasitol.

[11] Vuitton DA, McManus DP, Rogan MT, Romig T, Gottstein B, Naidich A, Tuxun T, Wen H, Menezes da Silva A (2020). International consensus on terminology to be used in the field of echinococcoses. Parasite.

[12] Deplazes P, Rinaldi L, Alvarez Rojas CA, Torgerson PR, Harandi MF, Romig T, Antolova D, Schurer JM, Lahmar S, Cringoli G, Magambo J, Thompson RC, Jenkins EJ (2017). Global distribution of alveolar and cystic echinococcosis. Adv Parasitol.

[13] Tamarozzi F, Akhan O, Cretu CM, Vutova K, Akinci D, Chipeva R, Ciftci T, Constantin CM, Fabiani M, Golemanov B, Janta D, Mihailescu P, Muhtarov M, Orsten S, Petrutescu M, Pezzotti P, Popa AC, Popa LG, Popa MI, Velev V, Siles-Lucas M, Brunetti E, Casulli A (2018). Prevalence of abdominal cystic echinococcosis in rural Bulgaria, Romania, and Turkey: a cross-sectional, ultrasound-based, population study from the HERACLES project. Lancet Infect Dis.

[14] Moro P, Schantz PM (2009). Echinococcosis: a review. Int J Infect Dis.

[15] Akgül H, Tez M, Unal AE, Keşkek M, Sayek I, Ozçelik T (2003). Echinococcus against cancer. why not? Cancer.

[16] Oikonomopoulou K, Yu H, Wang Z, Vasiliou SK, Brinc D, Christofi G, Theodorou M, Pavlou P, Hadjisavvas A, Demetriou CA, Kyriacou K, Diamandis EP (2016). Association between Echinococcus granulosus infection and cancer risk - a pilot study in Cyprus. Clin Chem Lab Med.

[17] Guan W, Zhang X, Wang X, Lu S, Yin J, Zhang J (2019). Employing parasite against Cancer: a lesson from the Canine Tapeworm Echinococcus Granulocus. Front Pharmacol.

[18] Nakao M, Sako Y, Yokoyama N, Fukunaga M, Ito A (2000). Mitochondrial genetic code in cestodes. Mol Biochem Parasitol.

[19] Donati S, Ciuffi S, Brandi ML (2019) Human circulating miRNAs real-time qRT-PCR-based analysis: an overview of endogenous reference genes used for data normalization. Int J Mol Sci 20(18). 10.3390/ijms20184353 10.3390/ijms20184353 PMC6769746 31491899

[20] Kosmider B, Wojcik I, Osiecka R, Bartkowiak J, Zyner E, Ochocki J, Liberski P (2005). Enhanced P53 and BAX gene expression and apoptosis in A549 cells by cis-Pt(II) complex of 3-aminoflavone in comparison with cis-DDP. Invest New Drugs.

[21] Baysal İ, Örsten S (2021). [Evaluation of the Effect of Hydatid Cyst Fluid on the apoptosis pathway in BEAS-2B and A549 cell lines]. Mikrobiyol Bul.

[22] Monteiro KM, de Carvalho MO, Zaha A, Ferreira HB (2010). Proteomic analysis of the Echinococcus granulosus metacestode during infection of its intermediate host. Proteomics.

[23] Chemale G, van Rossum AJ, Jefferies JR, Barrett J, Brophy PM, Ferreira HB, Zaha A (2003). Proteomic analysis of the larval stage of the parasite Echinococcus granulosus: causative agent of cystic hydatid disease. Proteomics.

[24] Aziz A, Zhang W, Li J, Loukas A, McManus DP, Mulvenna J (2011). Proteomic characterisation of Echinococcus granulosus hydatid cyst fluid from sheep, cattle and humans. J Proteom.

[25] Anderson NL, Anderson NG (2002). The human plasma proteome: history, character, and diagnostic prospects. Mol Cell Proteomics.

[26] Jacobs JM, Adkins JN, Qian WJ, Liu T, Shen Y, Camp DG, Smith RD (2005). Utilizing human blood plasma for proteomic biomarker discovery. J Proteome Res.

[27] Aref N, Shirzad H, Yousefi M, Yousofi Darani H (2013) Effect of different hydatid cyst molecules on Hela and Vero Cell lines growth in vitro. J Immunodeficiency Disorders 02. 10.4172/2324-853X.1000105

[28] Karadayi S, Arslan S, Sumer Z, Turan M, Sumer H, Karadayi K (2013). Does hydatid disease have protective effects against lung cancer?. Mol Biol Rep.

[29] Perlman H, Zhang X, Chen MW, Walsh K, Buttyan R (1999). An elevated bax/bcl-2 ratio corresponds with the onset of prostate epithelial cell apoptosis. Cell Death Differ.

[30] Yasen A, Wang M, Ran B, Lv G, Aji T, Xiao H, Shao Y, Wen H (2021). Echinococcus granulosus protoscoleces promotes proliferation and invasion of hepatocellular carcinoma cells. Cytotechnology.

[31] Orsten S, Baysal İ, Yabanoglu-Ciftci S, Ciftci T, Azizova A, Akinci D, Akyon Y, Akhan O (2021). MicroRNA expression profile in patients with cystic echinococcosis and identification of possible cellular pathways. J Helminthol.

[32] Ji J, Yamashita T, Wang XW (2011). Wnt/beta-catenin signaling activates microRNA-181 expression in hepatocellular carcinoma. Cell Biosci.

[33] Zhu W, Shan X, Wang T, Shu Y, Liu P (2010). miR-181b modulates multidrug resistance by targeting BCL2 in human cancer cell lines. Int J Cancer.

[34] Wang B, Hsu SH, Majumder S, Kutay H, Huang W, Jacob ST, Ghoshal K (2010) TGFbeta-mediated upregulation of hepatic miR-181b promotes hepatocarcinogenesis by targeting TIMP3. Oncogene 29(12) 1787-97. 10.1038/onc.2009.468 10.1038/onc.2009.468 PMC2845743 20023698

[35] Örsten S, Çiftçi T, Azizova A, Yüce G, Uysal A, İmamoğlu Ç, Karaağaoğlu E, Akıncı D, Akyön Y, Casulli A, Akhan O (2020). Investigation of the relationship between CE cyst characteristics and genetic diversity of Echinococcus Granulosus Sensu Lato in humans from Turkey. Parasitology.

[36] Schneider R, Gollackner B, Schindl M, Tucek G, Auer H (2010). Echinococcus canadensis G7 (pig strain): an underestimated cause of cystic echinococcosis in Austria. Am J Trop Med Hyg.

[37] Sadjjadi SM, Mikaeili F, Karamian M, Maraghi S, Sadjjadi FS, Shariat-Torbaghan S, Kia EB (2013). Evidence that the Echinococcus granulosus G6 genotype has an affinity for the brain in humans. Int J Parasitol.

